# A Simple Method of Treating Opium Poisoning

**Published:** 1897-02

**Authors:** 


### A SIMPLE METHOD OF TREATING OPIUM POISONING.

In country practice a medical man might possibly find himself very much embarrassed in presence of a case of opium poisoning through not having at hand the necessary drugs. A French contemporary, the *Medicine Moderne*, reports a case, however, in which a practitioner conceived the idea of giving subcutaneous injections of very strong coffee. On being called to the patient, a child of 10, who had accidentally been poisoned by paregoric elixir, it was evident that the case was very urgent, pupils imperceptible, only two or three respirations per minute, coma. It was impossible to administer internal remedies, and quite impracticable to wash out the stomach, as the accident had happened in the open country. Accordingly the practitioner ordered a very strong decoction of coffee to be made (equal parts of coffee and of water) and injected thirty drops hypodermically every ten minutes. After the fourth injection the breathing became freer, and the pulse fuller and more regular. In six hours the child was out of danger. The method is certainly one that is well worthy of being remembered and tried, especially by country practitioners, as opium poisoning is relatively a frequent accident among children, and is one that necessitates prompt and energetic measures on the part of the medical man.—*Medical Press and Circular.*

